# Recent advances in understanding and managing myasthenia gravis

**DOI:** 10.12688/f1000research.15973.1

**Published:** 2018-10-31

**Authors:** Allison Jordan, Miriam Freimer

**Affiliations:** 1 Department of Neurology, The Ohio State Wexner Medical Center, Columbus, Ohio, USA

**Keywords:** myasthenia gravis, autoantibodies, neuromuscular junction disorders, Eculizumab, Rituximab

## Abstract

Autoimmune myasthenia gravis (MG) is a neuromuscular junction disorder marked clinically by fatigable muscle weakness and serologically by the presence of autoantibodies against acetylcholine receptors (AChRs), muscle-specific kinase (MuSK), or lipoprotein-related protein 4 (LPR4). Over the past few decades, the mortality of patients with MG has seen a dramatic decline secondary to evolving interventions in critical care and medical management. In the past 2 to 3 years, there have been several changes in standard of care for the treatment of MG. These changes include confirmation of the benefit of thymectomy versus medical management alone in AChR patients and a new US Food and Drug Administration-approved medication for refractory MG. There are also several exciting new prospective drugs in the pipeline, which are in different stages of clinical trial testing.

## Introduction

Autoimmune myasthenia gravis (MG) is a neuromuscular junction (NMJ) disorder marked clinically by fatigable muscle weakness and serologically by the presence of autoantibodies. Autoantibodies against acetylcholine receptors (AChRs), muscle-specific kinase (MuSK), and lipoprotein-related protein 4 (LPR4) have been proven to be pathogenic ^1^. Several other antibodies such as agrin, cortactin, fast troponin, ryanodine receptor, and myofibrillar proteins have been discovered but were not able to induce the MG phenotype ^2^. The pathophysiology of the disease is dependent on the type of autoantibody present. In AChR MG, which accounts for about 85% of the population of patients with MG, IgG1 and IgG3 predominate ^3^. These antibodies bind directly and cause selective degradation of the receptors ^4^. Importantly, these immunoglobulins also cause activation of the complement pathway, including the membrane attack complex. Complement activation has been implicated as the major destructor of the neuromuscular endplate and has been observed in both human and animal models of MG ^5– 7^. In MuSK MG, which accounts for about 10% of the population of patients with MG, antibodies bind to the Ig-like region, blocking activation of the agrin–LRP4–MuSK complex and inhibiting neuromuscular transmission ^8^. Interestingly, the MuSK antibody is composed mostly of the IgG4 subtype, which does not have a predilection for activation of the complement cascade ^9^. LRP4 is a transmembrane protein, which functions as a receptor ^10^. Agrin binds LRP4, forming a complex that leads to MuSK activation. This activation appears to be essential for NMJ formation, including the distribution or clustering of the AChR ^10^.

The incidence of MG in the total population is rare; rates are estimated to be 5 to 30 cases per million person-years, and the prevalence of the disease is estimated to be 10 to 20 cases per 100,000 population ^11^. The annual average health-care cost in the US is estimated to be $20,190 per person ^12^, showing that although MG is rare, it can present a significant and chronic financial burden to those who carry the diagnosis. The mortality of those who carry a diagnosis has been decreasing ^13^, and this can be attributed to continued medical advancements, including better treatment options as well as improvements in acute critical care. Current treatment for MG includes anti-acetylcholinesterase (pyridostigmine) for daily or chronic symptom control; immunomodulatory therapies (intravenous immunoglobulin [IVIG] and plasma exchange), which are typically used for acute exacerbation of disease but have also been used for chronic symptom control; and immunosuppressant medications (steroids, azathioprine, cyclosporine, mycophenolate, and methotrexate), which are used for maintenance therapy and typically take weeks to months to see effect. It should be noted that of the above-listed agents, only IVIG has demonstrated clear efficacy in randomized, double-blind controlled studies ^14^. All other agents have failed to show significant improvement over placebo ^15– 17^. In the past 2 to 3 years, the standard of care for the treatment of MG has undergone several changes. The objectives of this article are to outline the most important advancements in care and to discuss new treatments in the pipeline.

## Recent changes in the treatment of myasthenia gravis

### Thymectomy

In 2016, the first randomized trial comparing thymectomy with medical management in patients with non-thymomatous MG was published ^18^. Although thymectomy in all patients (ocular and generalized) with AChR-positive MG with known thymoma was standard of care prior to the above publication, only observational and retrospective studies with conflicting conclusions had been published regarding the care of patients with non-thymomatous MG ^13, 19^. The patient population consisted of patients with a Myasthenia Gravis Foundation of America clinical classification of II to IV (indicating at least some generalized symptoms), AChR-positive MG, age of 18 to 65 years, and disease duration of 3 to 5 years. The range of disease duration reflects a change in inclusion criteria during the course of the study. It is important to note that patients with MuSK or LRP4 antibodies were not included in this study. Patients were randomly assigned to thymectomy plus prednisone or prednisone alone. The primary endpoints of the study were the quantitative MG (QMG) score and the required dosage of prednisone over the course of a 3-year period. Results showed that patients randomly assigned to the thymectomy group did better clinically over the 3-year period, with a mean average improvement of almost 3 points (2.85) on the QMG score. Patients in this cohort also required a lower prednisone dose over the 3-year period. There were also no treatment-associated complication differences between the two groups. This study gives good evidence for thymectomy in all generalized AChR-positive MG patients, regardless of thymoma status on imaging.

### Eculizumab

Eculizumab is a C5 monoclonal antibody directed at the complement protein C5 to prevent the formation of the terminal complement complex, C5b-9. It was previously approved by the US Food and Drug Administration (FDA) for paroxysmal nocturnal hemoglobinuria and atypical hemolytic uremic syndromes. The rationale for using this drug in MG stems from both human and animal studies showing complement-mediated damage at the NMJ endplate in MG, as described above ^6, 7^. In 2017, eculizumab was approved for MG on the basis of the REGAIN study ^20^. Patients enrolled in this double-blind placebo study had refractory generalized AChR MG, defined by the international consensus guidance for management of MG as unchanged or worsening post-intervention status after corticosteroids and at least two other immunosuppressive agents used in adequate doses for an adequate duration ^21^. The study looked at the change from baseline compared with placebo at week 26 of patient-administered quality-of-life and activity-of-daily-living surveys as well as physician-administered scoring systems. Although the study showed improvement from baseline in all administered scoring systems, only two (the QMG test and the MG-QOL15 survey) were statistically significant. Decreased MG exacerbations, need for rescue medications, and admissions to the hospital occurred in the patient population receiving drug as compared with placebo. The most common adverse effects were headache, upper respiratory tract infections, and nasopharyngitis. Eculizumab is known to place patients at a 1,000- to 2,000-fold greater risk for meningococcal diseases, and vaccination prior to starting the medication is recommended. Although the addition of a new FDA-approved medication for MG is exciting, it is important to remember that this medication should be reserved for patients with disease refractory to first-line treatments.

### Rituximab

There have been several prospective, retrospective, or case series published which suggest benefit with rituximab in patients with refractory MG ^22– 24^. Unfortunately, these studies had small populations of patients and lacked randomization and evaluator blinding. In 2017, a large multicenter blinded review was published comparing MuSK-positive MG patients who received rituximab with those who received other immunosuppressive medications (labeled as the control group) ^25^. This study enrolled 55 MuSK-positive MG patients. The primary endpoint was a Myasthenia Gravis Status and Treatment Intensity (MGSTI) score of 2 or more. Results of this review showed that patients treated with rituximab did meet the end goal of an MGSTI score of 2 or more, which was statistically significant when compared with the control group. The review did not show a decrease in hospitalizations across the two groups. Although this blinded review mimics enrollment in a randomized clinical trial, the data were collected retrospectively and this is a major limitation of this study. The BeatMG study, a randomized, double-blind, placebo-controlled phase 2 clinical trial looking at the safety and utility of rituximab, is currently in review. Participants with antibody-positive AChR (age range of 21 to 90 years) were randomly assigned to receive either rituximab or placebo (in addition to the patient’s baseline immunotherapy regimen). Primary outcomes looked at steroid requirement over the 52-week time period in addition to the safety profile of the drug ^26^. Preliminary data presented at the American Academy of Neurology in 2018 were not promising, as the study did not meet statistical significance in its primary endpoints. Final publication of this study’s results is pending.

## New drugs in the pipeline

Rozanolixizumab (UCB7665) is a humanized anti-human neonatal Fc receptor (FcRn) monoclonal antibody designed to reduce the levels of pathogenic IgG in autoimmune diseases. In prior studies, the drug was found to effectively reduce IgG in cynomolgus monkeys and was found to be safe in humans in a phase 1 study ^27^. Rozanolixizumab is currently in phase II trials for both MG and primary immune thrombocytopenia. In MG, this phase II trial will look at the effectiveness and safety of the drug as compared with placebo in patients with moderate to severe MG (ClinicalTrials.gov Identifier: NCT03052751).

Efgartigimod (ARGX-113), an FcRn monoclonal antibody, recently completed its phase II trial and will be continuing in a phase III study. Results of the phase II trial report that 75% of subjects had clinical improvement in MG activities of daily living (MG-ADL) scores in the 6-week period compared with 25% of placebo, with reduction of total IgG levels, and adequate tolerability. No severe or adverse events were reported during the study period ^26^.

Monarsen (EN101) is an antisense oligonucleotide which intermixes with the mRNA encoding for acetylcholinesterase, causing a reduction in production of the enzyme. A phase Ib, non-placebo-controlled, open-label study was completed in 2007. This study showed an improvement in QMG score of 87% of the participants (13 out of 15 participants), and no major adverse events were reported ^28^. In a phase II trial, 31 patients (23 of whom completed the study) showed improvement in QMG score while on the drug ^29^. Four adverse events were reported and one of these was death by cerebral hemorrhage. The study group did not think that these adverse events were related to the drug itself. There are no plans for further studies at this time.
